# Feedback-based noise management matrix in action

**DOI:** 10.4102/sajcd.v67i2.678

**Published:** 2020-04-01

**Authors:** Nomfundo F. Moroe, Katijah Khoza-Shangase, Milka Madahana, Otis Nyandoro

**Affiliations:** 1 Department of Speech Pathology and Audiology, Faculty of Humanities, School of Human and Community Development, University of the Witwatersrand, Johannesburg, South Africa; 2 Centre for Systems and Control Research, Faculty of Engineering, University of the Witwatersrand, Johannesburg, South Africa

**Keywords:** Complex intervention, Feedback-based noise monitoring model, Hearing conservation programme, Implementation, Mining, noise-induced hearing loss, Occupational noise, Risk management

## Abstract

**Background:**

This article presents the details and findings of a practical implementation of a preliminary model for use in hearing conservation programmes (HCPs) in the mining sector in South Africa.

**Objectives:**

The implementation is based on a previously published model, called feedback-based noise monitoring model (FBNMM), and represents its implementation as a tool of predicting occupational noise-induced hearing loss (ONIHL), as well as monitoring and managing HCPs in the mining sector within the South African context.

**Method:**

The article, utilising real miners’ data, demonstrates this basic static feedback model with its practical applications such as estimating, monitoring and providing quantitative information to aid miners, mining administrators and policymakers in decision-making around HCPs, as recommended in the previous 2019 publication by Moroe et al. This study was conducted in a South African platinum mine. Evidence on the model’s sensitivity and practicability in early identification, intervention and management of ONIHL in the workplace is presented.

**Results:**

Findings show how the use of the model within an HCP viewed as a complex intervention can allow for early prediction of ONIHL, consequently affording more accurate early intervention as part of preventive audiology within the health and safety goals of mines.

**Conclusion:**

The feedback-based model should be a useful tool for successful implementation and monitoring of HCPs within South African mines.

## Introduction

Occupational noise-induced hearing loss (ONIHL) is a complex disease (Le, Straatman, Lea, & Westerberg, 2017), which is recorded to be prevalent in the mining industry, with evidence suggesting that it is a leading work-related disability (Ritzel & McCrary-Quarles, 2008). Ritzel and McCrary-Quarles (2008) assert that, second only to presbycusis, ONIHL is a form of acquired hearing loss that presents the sufferer with significant negative consequences following exposure to high levels of noise (Ritzel & McCrary-Quarles, 2008). Regardless of the fact that hearing loss has been considered as non-life-threatening, the presence of it, even in mild forms, is sufficient evidence to prove that if untreated it can lead to profound psychological and psychosocial consequences for the sufferer’s quality of life (Tye-Murray, 2009). Furthermore, additional negative consequences of prolonged exposure to hazardous noise have been published, and these include increased fatigue and decreased concentration, which are known to increase human errors at work (Amjad-sardrudi, Dormohammadi, Golmohammadi, & Poorolajal, 2012; Moroe, Khoza-Shangase, Madahana, & Nyandoro, 2019; Picard et al., 2008).

As far as the influence of ONIHL on the employee’s ability to execute or complete tasks that depend on auditory signals or verbal communication, evidence suggests negative outcomes for employees with ONIHL (Thorne, 2006). In addition, the presence of ONIHL, which has a direct link to the employee’s communication handicap, may lead to workers being regarded as incompetent or inactive in their job, which eventually impacts negatively on teamwork and team productivity (Momm & Geiecker, 2009). This impact on teamwork extends to negative influences on communication amongst workers, which impacts not only the workers’ ability to communicate amongst each other, but also on their ability to observe safety standards. Employees with ONIHL may be unable to hear and respond to warning and safety signals such as alarms that are often in high frequencies, which are often in high-frequency sounds that are the most affected in ONIHL. There is also the argument that tinnitus affects the quality of life, which could contribute to an increase in human errors and ultimately to an increase risk of accidents in this population (Kirchner et al., 2012).

Thorne (2006) highlights an additional impact of ONIHL, which centres around employment opportunities and career options and advancement that a hearing impaired individual will experience. This additional limitation raises important economic burdens for both the individual affected and the state, particularly in low- and middle-income countries like South Africa. The fact that ONIHL contributes to occupational injuries because of the employee’s inability to hear warning sirens, ill health and its subsequent absenteeism for various reasons, several authors argue that ONIHL leads to significant social and economic implications for everyone involved, including the employee, their families, communities and the state as a whole (Amjad-sardrudi et al., 2012; Coderio, Clementa, Diniz, & Dias, 2005; Hermanus, 2007; Kramer, Kapteyn, & Houtgast, 2006; Moroe et al., 2019).

Various risk factors have been documented for the development of hearing loss. These risk factors explain why individuals exposed to the same noise levels respond differently (Daniel, 2007; Sliwinska-Kowalska et al., 2005; Sliwinski-Kowalska & Davis, 2012). Evidence suggests that these risk factors make an individual more susceptible to ONIHL than another. Daniel (2007) categorises these risk factors under two groups: (1) modifiable factors, which fall within an individual’s control, for example, variables such as smoking and exposure to drugs that are known to lead to ototoxicity such as anti-tuberculosis, antiretroviral and anti-cancer drugs (Khoza-Shangase, 2013, 2019); and (2) non-modifiable factors, which fall outside the individual’s control. The presence of these risk factors, singly or combinedly, exacerbates the susceptibility of an individual exposed to hazardous noise levels to ONIHL, with its consequent negative sequelae to health and safety.

Reduction and/or elimination of ONIHL in the mining sector has been linked to successful implementation of hearing conservation programmes (HCPs), where effective ways of assessing, monitoring and reducing or eliminating excessive workplace exposure to hazardous noise are adhered to (Amedofu, 2007; Chadambuka, Mususa, & Muteti, 2013; Feuerstein, 2001; Moroe, Khoza-Shangase, Kanji, & Nthlakana, 2018; Moroe et al., 2019). Efficacy of HCPs is increased where the hierarchy of control is implemented accordingly, although in the mining sector noise is ubiquitous as engineering controls are not yet well developed to eliminate noise completely. Efforts towards strategies to enhance the efficacy of HCPs are therefore important. Elsewhere, authors have presented a model called feedback-based noise monitoring model (FBNMM). The purpose of this model is to be utilised as a tool of managing ONIHL in the mining sector within the South African context. In Moroe et al. (2019), the authors describe how the model was conceptualised from a risk management framework that acknowledges that ONIHL is a risk that needs to be eliminated or reduced through effective management strategies (Moroe et al., 2019). The framework agrees with Donoghue’s (2001) assertion that anything presenting a threat to health and safety within an organisation, which may put employees at risk for injury or harm, requires systematic management. This is in line with what Hermanus (2007) argues when he states that in occupational health and safety:

[*T*]he underlying premise of risk management is that improvements in health and safety can be made by correctly identifying and addressing hazards or factors (which may be underlying or direct) that contribute to occupational risk. (p. 536)

## The feedback model

### Feedback model overview

**FIGURE 1:**
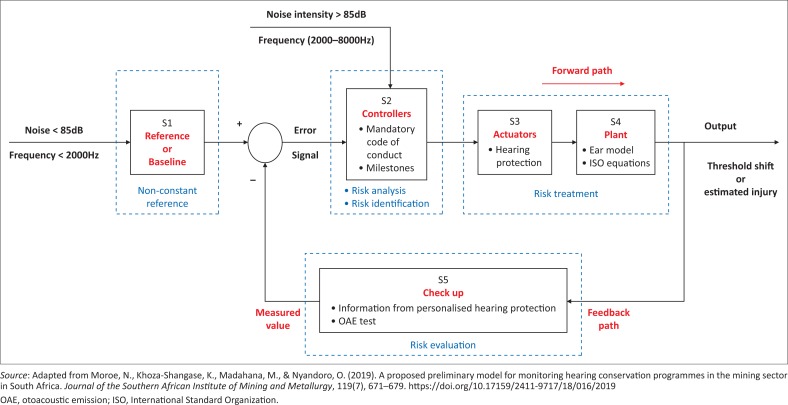
Enhanced feedback-based noise management matrix.

According to Madahana, Ekoru and Nyandoro (2019):

From Figure 1, a mining employee represented by (s4) working in an environment with noise intensity equal or greater than 85 dB is expected to adhere to the mandatory code of practice, summed up by the noise policy in the mines as shown in (s2). One of the policies requires the […] employees to wear hearing protection (s3). The employee undergoes periodic hearing check-up (s5) conducted annually or biannually. For our system, it is proposed that the check-up should be done at a much shorter interval for instance daily. The authors are cognizant that daily tests will require significant interventions and buy-in as this move has implications for production time as well as changes to behavioural aspects, all-inclusive of mental models that would require adjustments. Additionally, this will have financial implications for staff, equipment and maintenance of the testing facilities; including transportation, since medical centres are not always available on site. The information obtained from the check-up is compared to the base line or reference (s1). If the employee has experienced significant threshold shift from the baseline, then, the mining administrators should apply other policies and the whole process repeats again. For effective communication to happen between the employees and the administrators, the information on the current state of hearing (daily threshold shift), is sent to the administrator via a data processing unit. (p. 4)

### Improvements to the current model

The current model shown in Figure 1 has the following enhancements from the previous models by Moroe et al. (2019) and Madahana et al. (2019), which was further improved by Madahana, Ekoru and Nyandoro (in press):

- Subsystem 1, which is also the baseline or the reference, is interpreted as the tracking unit. It is a non-constant reference. With sufficient hearing protection, the mine worker’s threshold shift is expected to closely follow the baseline. This would then be interpreted that the miner is only being affected by presbycusis and not occupational noise.
- Actual values are used in the input noise intensity that is a disturbance to the system. The exposure levels for each mine worker are identified according to the drilling machine that the mine worker operates.
- To simplify the developed current model, the risk treatment is represented by the actuator (hearing protection) and the plant (ISO equations that represent hearing loss). These are also considered to be on the forward path.

### Feedback model validation

Data used for model validation were collected from all the individuals who work at the platinum mine. However, based on key findings by Edwards and Kritzinger (2012), when they conducted a study on NIHL milestones, their research findings indicate that when comparing mine workers for the average percentage loss hearing (PLH) in 1 year, for different occupations within the mines, the deep gold mine drillers were the worst. Edwards et al. (2012) and Wagner (2019) further explain that the deep gold mine drillers work in an environment that is at a depth of 2 km underground and up to 10 km on a vertical plane. The rock gold miners work on the rock face and their work hours usually exceed 8 h. They experience noise mostly from transportation, drilling equipment and ventilation. Furthermore, the underground spaces are restricted with poor acoustics that affect the mine workers (Phillips, Heyns & Nelson, 2007).

The current study therefore targeted one of the worst-case scenarios of mine employees. These were the workers who are affected the most by occupational noise because of the nature of their tasks in the mine. Edwards and Kritzinger (2012) proposed that in order for HCPs to be successful in ensuring zero harm (MHSC, 2014), they need to be commodity specific – and the current study’s commodity was platinum. These authors also recommend that HCPs must be mine-specific, and the current study was specific to one platinum mine in South Africa. Lastly, Edwards and Kritzinger (2012) argue that HCPs must be occupation-specific; and in the current study, for illustration of the FBNMM, drillers working in a deep platinum mine were chosen. The provided factors are not exhaustive with regard to the development of an effective HCP. Other factors, for instance, the maturity (size as well as the staffing and resource allocation) of the company, may impact the success of an HCP.

### Current hearing protection used in the platinum mine

In this study, the two forms of hearing protection used by the rock drill mine operators are described below:

1. 3M peltor H9A optime 98 over-the-Head earmuff. Noise reduction rating (NRR)*: 25 dB. CSA Class A. *The NRR may overestimate the hearing protection provided during typical use. 3M recommends reducing the NRR by 50% for estimating the amount of noise reduction provided (3M South Africa, 2019).
2. Personalised hearing protection with the highest attenuation of 18.35 dBA and lowest attenuation of 7.1 dBA custom-made clippers (Kock, 2013) is sometimes used.

Consequently, the data shown in Table 1 were used to validate the model.

**TABLE 1:** Rock driller operators’ threshold shift for a deep platinum mine in South Africa.

Age	Years of service	Hours of work	Exposure levels in dBA	Attenuation at 25 dBA	Attenuation at 18.35 dBA	Baseline	Threshold shift from baseline
44	9	8	104	79	85.5	14.5	15.6
38	9	8	107	82	88.5	8.3	7.1
51	5	8	105	80	86.5	24.6	32.7
39	3	8	108	83	89.5	15	16.5
55	9	8	110	85	91.5	12.7	14

*Source:* Table created using data from Moroe, N.F. (2018). *Occupational noise-induced hearing loss in South African large scale mines: From policy formulation to implementation and monitoring*. Doctoral thesis. Johannesburg: University of the Witwatersrand.

Note: Age, the current age of the mine worker; Years of service, the number of years a mine worker has worked as a rock driller; Exposure levels, the measured noise intensity the mine worker is exposed to at a mining environment; Attenuation levels at 25 dBA, the expected reduction in exposure levels when wearing hearing protection with specification that indicate that the attenuation will be 25 dBA; Attenuation levels at 18 dBA, the expected reduction in exposure levels when wearing hearing protection with specification that indicate that the attenuation will be 18 dBA; Baseline, hearing threshold shift obtained from the audiogram when the mine worker started working with a company (the mine worker could have previously been employed in another mine); Threshold shift from baseline, the value by which the mine worker’s hearing has shifted from the baseline.

### Sample set of the input data

Table 1 provides the sample data set used to simulate the different scenarios in the mines.

#### Assumptions while exploring the data set provided

It is assumed that rock drill workers work for 8 h per day for 5 days (as per the report on the data set provided).

The mine workers are not only exposed to drilling noise but also to the different sources of noise within the mine, for instance, from transportation.

It is assumed that all rock drillers use the 18.35 dBA and 25 dBA attenuation setting provided and not the worst-case scenario of 7.1 dBA.

The data set did not indicate the exact hearing protection that the mine worker uses; hence, both case scenarios are simulated (i.e. the 25 dBA and 18.35 dBA).

#### Data collection method

Two hundred and ten rock drill operators employed in a platinum mine were chosen. Their occupational exposure histories were collected. Five variables which can influence hearing loss were used in the development of the predictive model.

### Ethical considerations

Ethical approval to conduct the study was obtained from the Human Research Ethics Committee of the University of the Witwatersrand (Medical; Protocol number M160264) on 18 April 2016. Data used in the current study were obtained from a bigger study entitled ‘Occupational noise-induced hearing loss in South African large-scale mines: From policy formulation to implementation and monitoring’ (Moroe, 2018). The data were collected through retrospective document review of the mine. All procedures contributing to this work complied with the relevant national and institutional guidelines for research on human subjects and adhered to the Declaration of Helsinki 1975 as revised in 2008.

## Results and discussion

**FIGURE 2:**
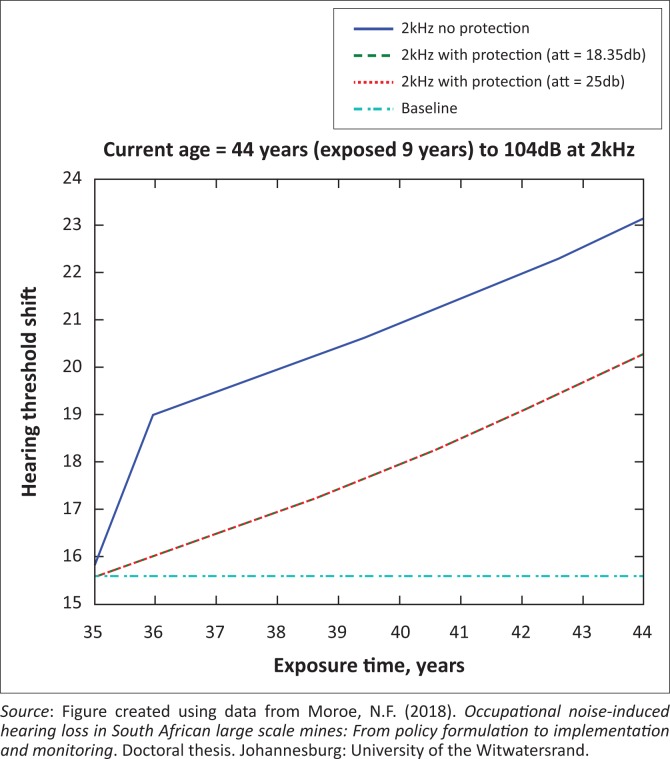
Forty-four years old at 2000 Hz with exposure levels of 104 dB.

The presented results in Figure 2 to Figure 16 are for five mine workers, each with a unique baseline and threshold shift. The mine workers operate different types of drilling machines, hence the variation in the exposure levels. The mine workers, however, are provided with the same hearing protection irrespective of the machine they are operating. The mine workers use both the earmuffs (3M Science South Africa, 2019) and personalised hearing protection custom-made noise clippers (Kock, 2013). Therefore, the presented results show how the mine workers’ threshold shifts would change for both types of hearing protection. For each mine worker, three case scenarios of the attenuation levels provided are investigated using the FBNMM. The feedback model is a predictive tool that can be used as part of the HCP to predict the future threshold shift of mine workers, which then affords the mining administrators an opportunity to provide early intervention for preventive care.

**FIGURE 3:**
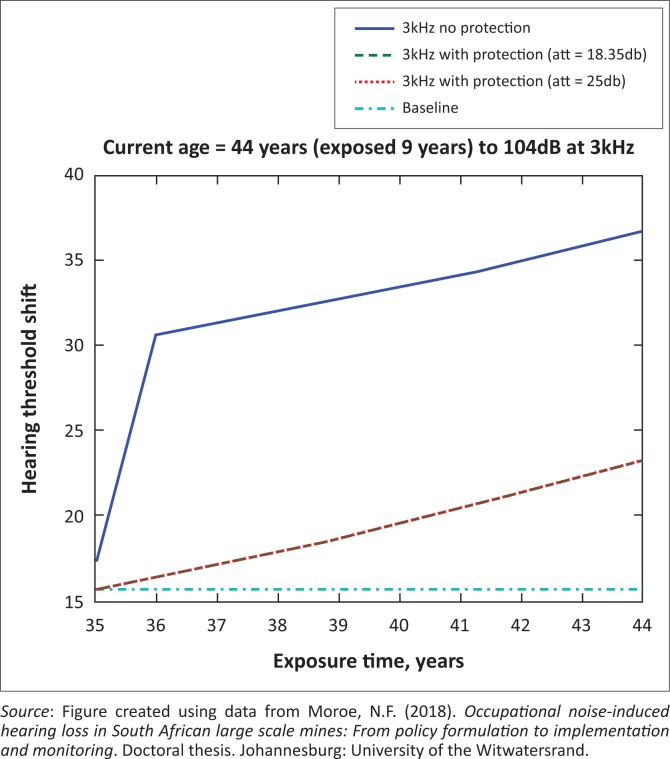
Forty-four years old at 3000 Hz with exposure levels of 104 dB.

**FIGURE 4:**
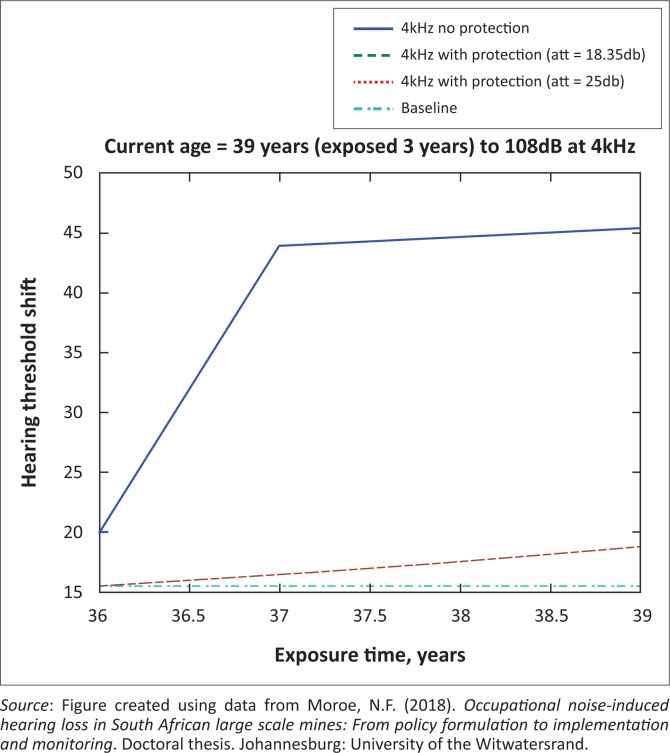
Thirty-nine years old at 4000 Hz with exposure levels of 108 dB.

**FIGURE 5:**
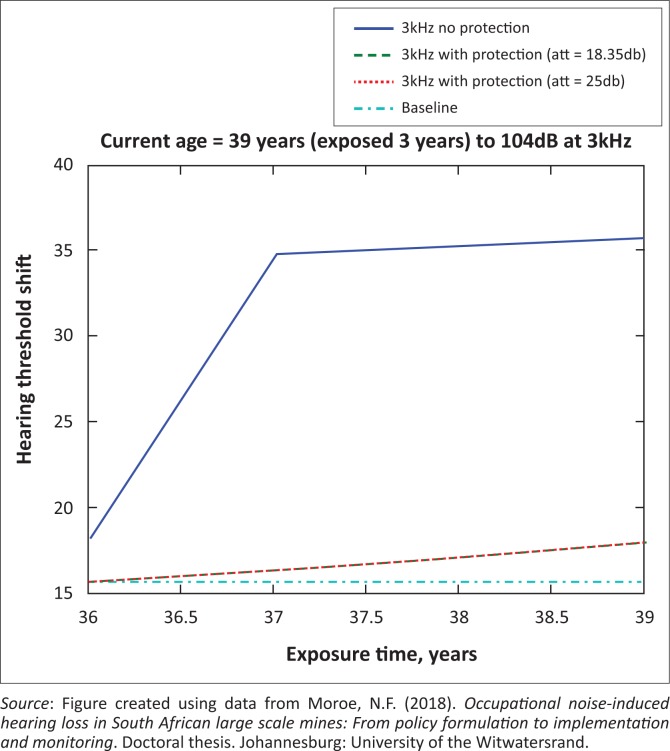
Thirty-nine years old at 3000 Hz with exposure levels of 108 dB.

**FIGURE 6:**
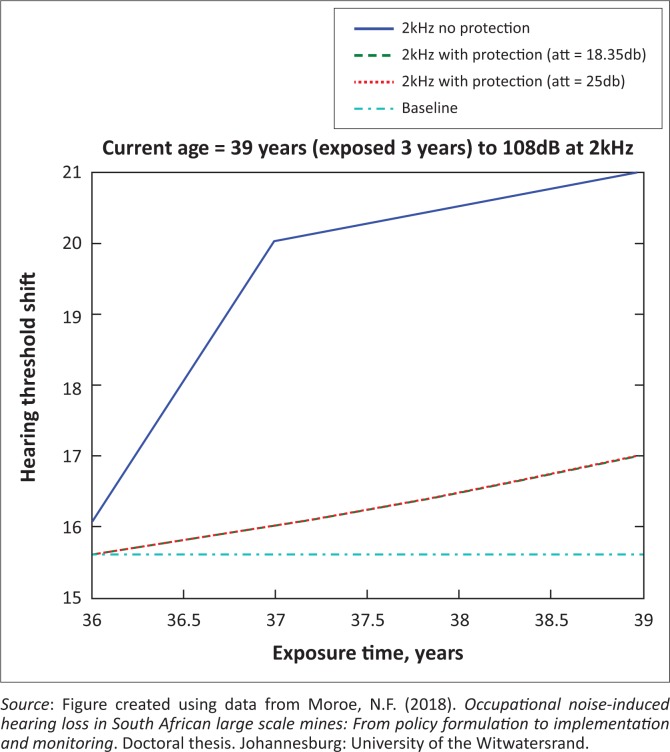
Thirty-nine years old at 2000 Hz with exposure levels of 108 dB.

**FIGURE 7:**
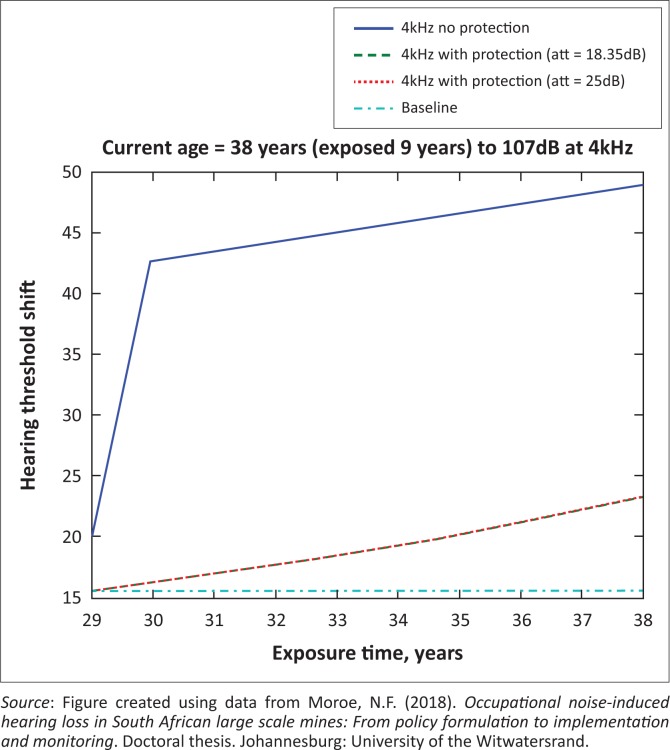
Thirty-eight years old at 4000 Hz with exposure levels of 107 dB.

**FIGURE 8:**
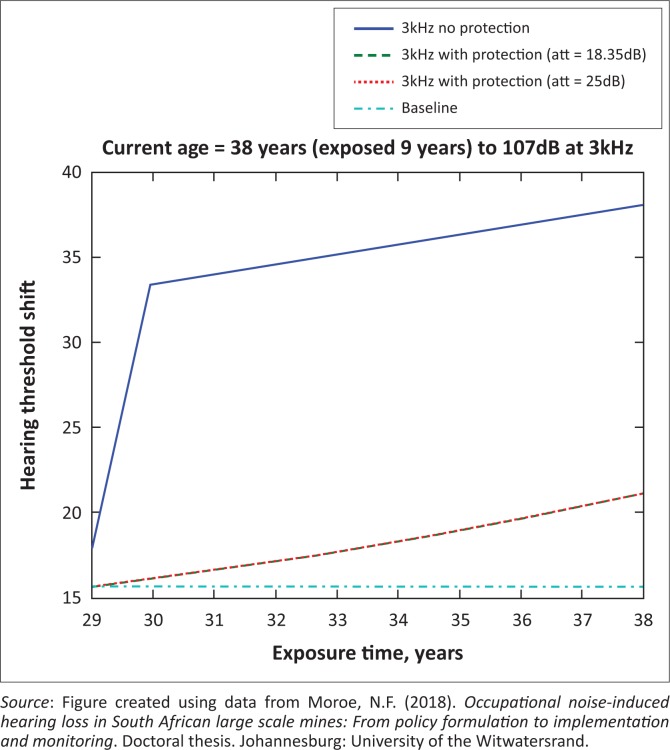
Thirty-eight years old at 3000 Hz with exposure levels of 107 dB.

**FIGURE 9:**
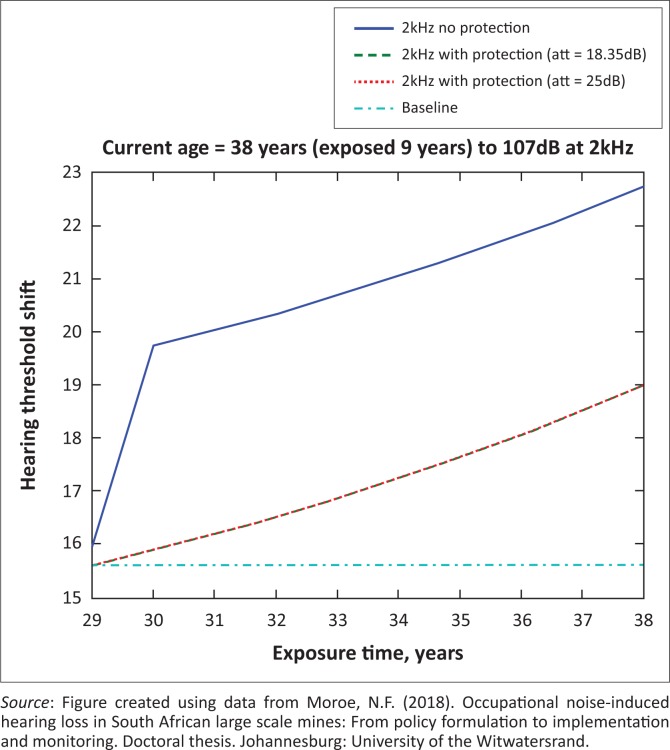
Thirty-eight years old at 2000 Hz with exposure levels of 107 dB.

**FIGURE 10:**
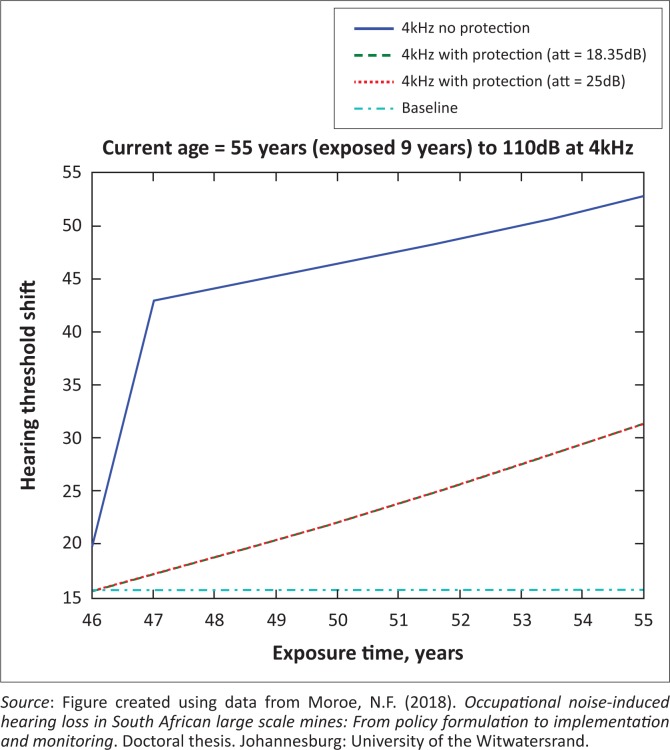
Fifty-five years old at 3000 Hz with exposure levels of 110 dB.

**FIGURE 11:**
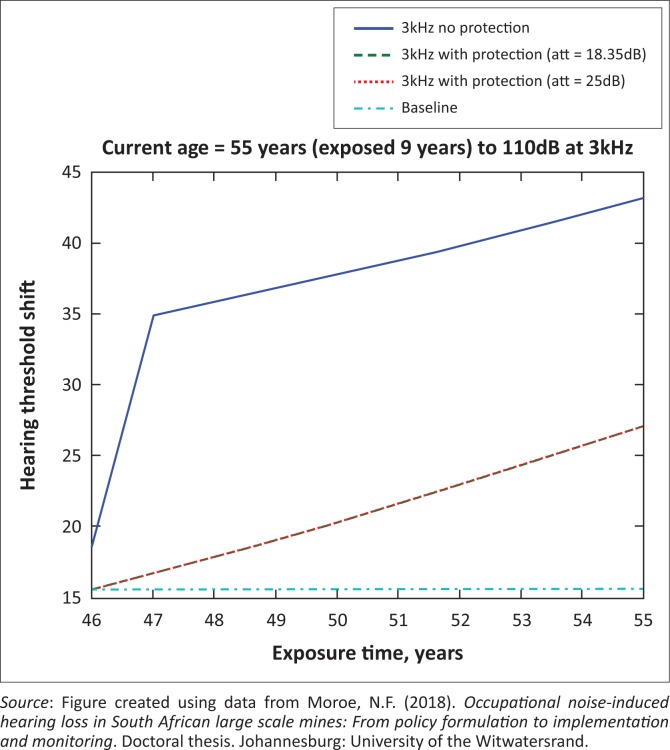
Fifty-five years old at 3000 Hz with exposure levels of 110 dB.

**FIGURE 12:**
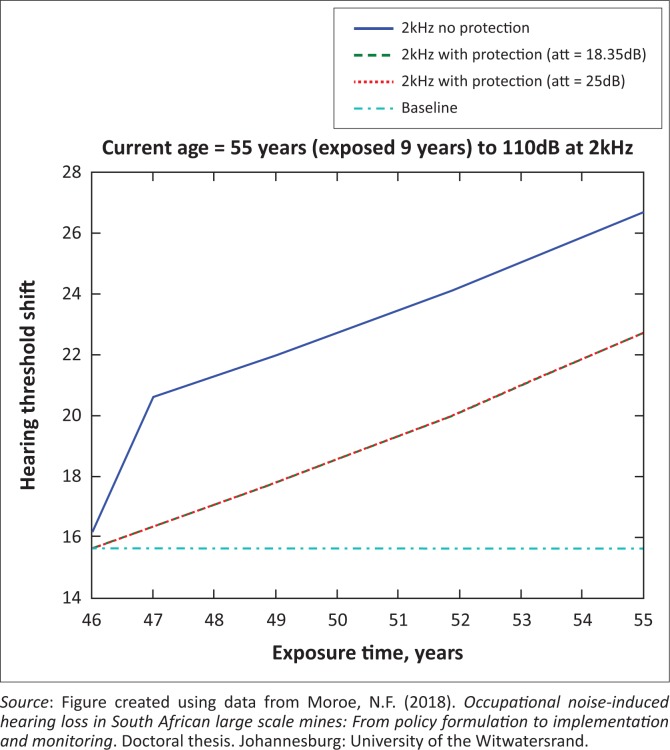
Fifty-five years old at 2000 Hz with exposure levels of 110 dB.

**FIGURE 13:**
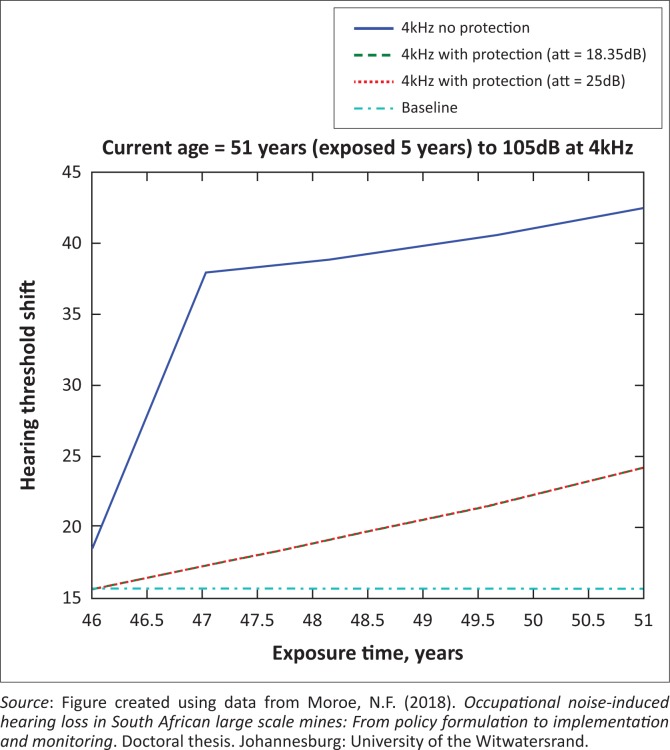
Fifty-one years old at 4000 Hz with exposure levels of 105 dB.

**FIGURE 14:**
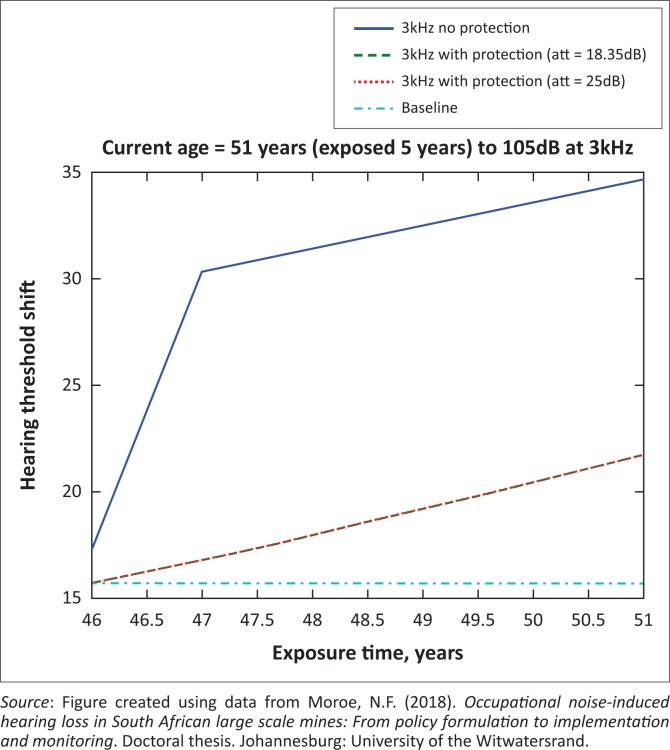
Fifty-one years old at 3000 Hz with exposure levels of 105 dB.

**FIGURE 15:**
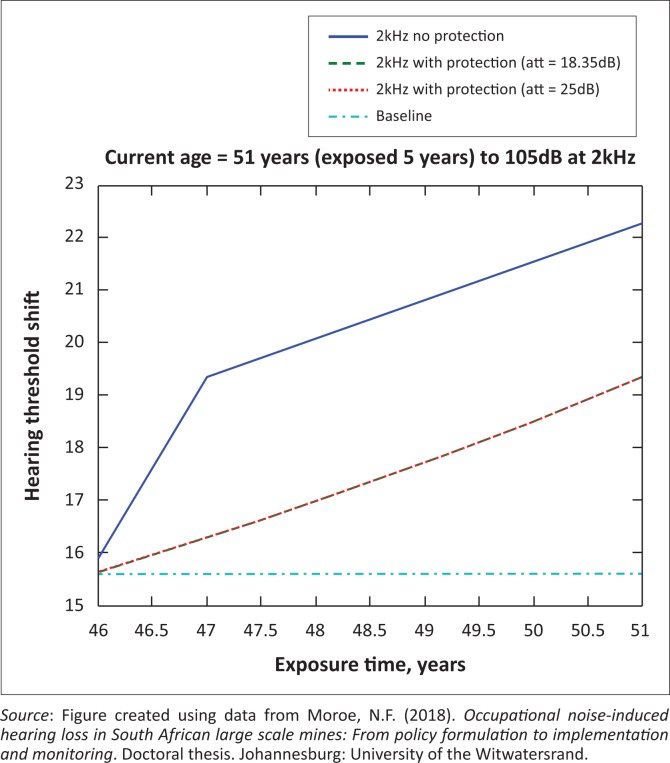
Fifty-one years old at 2000 Hz with exposure levels of 105 dB.

**FIGURE 16:**
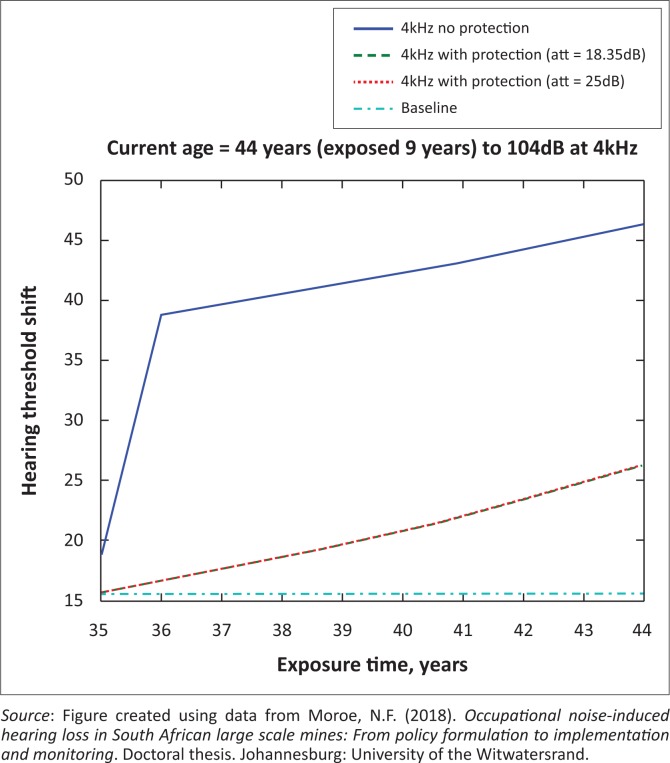
Forty-four years old at 3000 Hz with exposure levels of 105 dB.

### Graphs that use the feedback-based noise monitoring model to predict hearing loss using historical data

Figure 2 to Figure 16 represent a plot of the hearing threshold versus exposure time in years at specified frequencies (2000 Hz, 3000 Hz, 4000 Hz), and specific exposure levels in decibels for each mine worker. The speech frequencies are used to predict how a mine worker’s thresholds would change for given attenuation. The graphs indicate the prediction of the threshold shift if the mine worker does not wear hearing protection, if the mine worker wears earmuffs or if the mine worker wears personalised hearing protection. The baseline for the mine workers was obtained from their audiograms as well as their years of employment in this particular mine. Although some of the mine workers do have previous history of employment, the baseline used in the current study is the one that was obtained when the mine worker started employment at the mine specific to this study.

### Deductions from the predictive tool and the audiograms of the mine workers

The predictive tool using the provided inputs presents a futuristic estimation of how the thresholds will shift as shown in Figure 2 to Figure 16. Current predictions reveal that as the platinum mine employed the predictive tool at the beginning of the careers of the five miners used as examples in this study, clear indications of future hearing loss are illustrated (Table 2); and most importantly, ONIHLs with the type of hearing protection provided by the mine are found. This prediction assumes that the mine worker consistently and appropriately wears their hearing protection at all times when exposed to occupational noise. The prediction also assumes that there are no additional toxins, such as ototoxicity, in the miners’ medical history.

**TABLE 2:** Actual and predicted hearing using the feedback model.

Age	Years of service	Actual	Predicted after years of service
44	9	Mild to moderately severe	Slight hearing loss
38	9	Slight	Normal hearing loss
51	5	Mild to moderate hearing loss	Mild to moderate hearing loss
39	3	Moderate	Slight hearing loss
55	9	Moderate	Slight to mild hearing loss

*Source:* Table created using data from Moroe, N.F. (2018). *Occupational noise-induced hearing loss in South African large scale mines: From policy formulation to implementation and monitoring*. Doctoral thesis. Johannesburg: University of the Witwatersrand.

In Table 2, the actual and predicted hearing loss is presented. Nuanced descriptive analysis shows that the older mine workers present with a greater increase in their hearing loss than the younger age groups; hence, other methods of interventions should be considered for them in the mine’s HCP. The mine administrators should consider, for example, reducing their hours of work or providing them with hearing protection with sufficient noise attenuation. Control measures can also be taken for this group, for example, investigating the *quiet* level of machines they use as well as the age of the machines being used. Although these strategies would be important for all mine workers, current findings suggest more active and age-specific interventions, and hence the suggestions made for the older miner in this article.

## Hearing protection

It is observed in Figure 2 to Figure 16 that attenuation at 18.5 dB and 25 dB is similar such that the two attenuations superimpose on each other. It was expected that the 25 dB would provide better attenuation of noise; however, from the manufacturer’s data sheet, it is stated that the earmuffs are recommended for time-weighted average noise exposures up to 98 dB. This is an important consideration for the current mine as the mine workers are exposed to noise intensity levels between 104 dB and 110 dB – significantly more than the 98 dB that the earmuffs were developed for. The implication of this is that the earmuffs currently used would not be able to actually provide an attenuation of 25 dB which would have been considered sufficient for an effective HCP in this mine.

The FBNMM that was put into action in this article clearly provided a prediction of how a mining employee’s hearing function would present with given attenuation at speech frequencies. Suppose that this tool had been employed at the beginning of the employee’s career, then correct measure to ensure zero harm as required by the Mine Health and Safety Council would have been taken by the mine administrators in the performance of administrative controls and personal hearing protection pillars of their HCP. The FBNMM is significant to the HCP because it is a tool that can be used to predict hearing impairment and allow for provision of early intervention to prevent ONIHL (Moroe et al. 2019).

This tool would, however, efficiently work if other significant factors that affect susceptibility of the platinum workers to ONIHL, such as burden of disease in the form of tuberculoses (TB) and HIV, the presence of dust, ototoxic chemicals and cigarette smoking, are considered and managed as part of the HCP. These factors, which were not added into the FBNMM as inputs, are argued as the possible explanation as to why there was an inability of the graphs to closely track (follow) the baseline irrespective of the hearing protection provided. Current implementation of the FBNMM reveals that, over and above what Edwards and Kritzinger (2012) list as key considerations for HCPs, HCPs should also be age-specific and baseline thresholds specific for accurate predictions and consequent efficient interventions to be provided. Furthermore, HCPs need to view ONIHL within this context to be complex, with many influencing factors, as evidenced by the slight differences in the actual and predicted results in the current study – which could only be accounted for by the lack of the FBNMM to include other inputs that may be significant in ONIHL.

## Recommendations and conclusion

Putting the FBNMM into action in this article yielded positive findings that have important implications for HCPs. Furthermore, actionalising the FBNMM raised the following recommendations for future studies, which will be tested, documented and reported on an already planned follow-up paper:

1. inclusion of more dynamic aspects to the system, for example, the use of an ear model of a human being
2. inclusion of other aspects that affect the mining environment, for instance, acoustic environment, temperature and ventilation in account during the period values were selected versus, how it would be in the future? Does it consider, for example, number of drills in operation at a time, or the acoustic environment variations machinery in operation, faults or dykes, water, etc.?
3. inclusion of other risk factors, over and above age, gender, ethnicity, nutrition, exposure to chemicals (in the workplace as well as because of medication), family history of exposure and the likelihood of developing ONIHL at a faster rate once exposed previously that influence susceptibility of an individual to ONIHL in the model
4. development of a preliminary working prototype to be used in monitoring of ONIHL in the mine
5. inclusion of artificial intelligence and machine learning concepts in the working prototype.

The use of metrics, as was performed in the current study, is argued by the authors to be advancement in the management of ONIHL and has significant value towards efficacious implementation and monitoring of HCPs. The close link of metrics with artificial intelligence places this advancement at the forefront of audiology’s engagement with the Fourth Industrial Revolution in combating ONIHL.
